# 

**Published:** 1829-02

**Authors:** 


### MONTHLY LIST OF MEDICAL BOOKS.

[*Medical Works cannot be entered on this List except a copy be sent for the purpose; the titles of Books having frequently been transmitted to us, as published, which have not appeared for weeks, or even months, after.*]

On Aneurism, and its Cure by a new Operation. Dedicated, by permission, to the King. By James Wardrop, Surgeon to his Majesty.—London: 1828. 8vo. pp. 117. Plates.

Transactions of the Medical and Physical Society of Calcutta. Vol. III. —Calcutta, 1827. 8vo. pp. 454.

An Essay on the Mechanism of Parturition. From the German of C. F. NAEGELE, Professor of Midwifery at Heidelberg. By EDWARD RIGBY, M.D.—12mo. pp. 166.

Naploeon à St. Hélène. Opinion d'un Medecin sur la Maladie de l'Empereur Napoleon, et sur la Cause de sa Mort. Par J. HEREAU.—Paris, 1829. 8vo. pp. 228.

A Letter to the Right Hon. the Secretary of State, containing Remarks on the Anatomical Report, and pointing out the Means by which the Science of Anatomy may be cultivated with advantage and safety to the Public. By G. J. GUTHRIE, F.R.S. Professor of Anatomy and Surgery to the Royal College of Surgeons, &c.—8vo. pp. 37.

Origin of Life and Cause of Diseases, clearly explained. By JAMES MORISON, the Hygeist.—Pp. 16.

### NOTICES.

*Communications have been received from* Dr. SPEER, Mr. CHINNOCK *and* Mr. G. T. BURNETT.

*In answer to two letters which have been addressed to them, the Editors beg to reply, that they have no objection to publish Original Papers anonymously; but the names of the Authors must always be confided to them.*

*If* Dr. M., *of Exeter, will forward a copy of his work to the Publisher's, the Editors will give it early attention. They have communicated with* Dr. MACLEOD *upon the subject, but he does not remember ever to have received the Dissertation.*

